# The Effects of *Rf4* and the Genetic Mechanism Behind Fertility Restoration of Wild Abortive Cytoplasmic Male Sterility (WA-CMS) in* Japonica* Rice (*Oryza sativa* ssp. *Japonica*)

**DOI:** 10.1186/s12284-022-00605-0

**Published:** 2022-11-28

**Authors:** Honggen Zhang, Xixu Li, Zuopeng Xu, Xiangqiang Zhao, Zihao Wan, Xiaojun Cheng, Qiaoquan Liu, Minghong Gu, Shuzhu Tang

**Affiliations:** 1 Jiangsu Key Laboratory of Crop Genetics and Physiology/Key Laboratory of Plant Functional Genomics of the Ministry of Education/Jiangsu Key Laboratory of Crop Genomics and Molecular Breeding, Agricultural College of Yangzhou University, Yangzhou, 225009 China; 2 Jiangsu Co-Innovation Center for Modern Production Technology of Grain Crops, Yangzhou University, Yangzhou, 225009 China; 3 School of Life Sciences, Nantong University, Nantong, 226019 China

**Keywords:** WA-type CMS, *Japonica* rice, Restorer-of-fertility gene (*Rf*), Fertility restoration, Cytoplasmic male sterility

## Abstract

Wild abortive-type cytoplasmic male sterility (WA-type CMS) has been exclusively used in hybrid seed production in *indica* rice cultivars, and fertility restoration in WA-type CMS is controlled by two major restorer genes, *Rf3* and *Rf4,* through a sporophytic mechanism. However, the genetic mechanism underlying fertility restoration in WA-type CMS in *japonica* cultivars is poorly understood. In the present study, C418, a leading Chinsurah Boro II- (BT)-type *japonica* restorer line, showed partial restoration ability in WA-type *japonica* CMS lines. The 1:1 segregation ratio of partially fertile to sterile plants in a three-cross F_1_ population indicated that fertility restoration is controlled by one dominant gene. Gene mapping and sequencing results revealed that the target gene should be *Rf4*. The *Rf4* gene restores fertility through a sporophytic mechanism, but the *Rf4* pollen grains show a preferential fertilization in the testcross F_1_ plants. Furthermore, *Rf4* was confirmed to have only a minor effect on fertility restoration in WA-type *japonica* CMS lines, and *Rf* gene dosage effects influenced the fertility restoration of WA-type CMS in *japonica* rice. The results of our study not only provide valuable insights into the complex genetic mechanisms underlying fertility restoration of WA-type CMS but will also facilitate the efficient utilization of WA-type CMS in *japonica* rice lines.

**Supplementary Information:**

The online version contains supplementary material available at 10.1186/s12284-022-00605-0.

## Background

Cytoplasmic male sterility (CMS), a maternally inherited trait, prevents the production of functional pollen grains and has been observed in more than 150 plant species (Fujii and Toriyama 2009). CMS can be suppressed by specific nuclear-encoded restorer-of-fertility (*Rf*) genes. CMS/*Rf* systems provide a useful genetic tool for the utilization of heterosis, and this breeding system is known as a three-line system, which is developed by using a CMS line, a maintainer line with the same nucleus as the CMS line, and a restorer line that contains the *Rf* gene(s). To date, hybrid seed production in many cultivated crop species is performed using the three-line hybrid breeding system (Zheng et al. 2020; Huang et al. 2015a, b; Melonek et al 2021).

Rice is a staple food for more than half of the world's population, and the development of new and effective agricultural technologies to increase rice production is essential to meet global food demands and to ensure food security. In the past 50 years, three-line hybrid breeding in rice has made a significant contribution to the increase in yield (Chen and Liu 2014; Huang et al. 2014; Li et al. 2007; Kim and Zhang 2018). In rice, over 20 types of CMS systems have been discovered, among which three representative CMS types, including the wild abortive (WA)-, Honglian (HL)-, and Chinsurah Boro II (BT)-types have been widely used for breeding (Chen and Liu 2014; Huang et al. 2014; Li et al. 2007). At present, WA-type and HL-type CMS have been used in three-line *indica* hybrid seed production, and BT-type CMS has used in the breeding of *japonica* hybrids for cultivation (Chen and Liu 2014; Huang et al. 2014; Li et al. 2007; Yuan 1994). Several *Rf* loci used for the breeding of restorer lines have been identified and mapped. *Rf3* and *Rf4*, two major fertility restorer genes for WA-type CMS, have been mapped to chromosomes 1 and 10, respectively (Ahmadikhah and Karlov 2006; Tang et al. 2014; Zhang et al. 1997). *Rf5* and *Rf6*, two major fertility restorer genes for HL-type CMS, have been mapped to chromosomes 10 and 8, respectively (Hu et al. 2012; Huang et al. 2000, 2012; Huang et al. 2015a, b). The *Rf1a*/*Rf1b* genes at the *Rf1* locus of BT-type CMS have been mapped to chromosome 10 (Akagi et al. 1996; Komori et al. 2004; Wang et al. 2006). With the exception of *Rf3*, all of these *Rf* genes have been cloned. Male fertility in rice plants carrying WA-type cytoplasm is restored sporophytically, while male fertility of rice plants carrying the BT- and HL-type cytoplasm is restored gametophytically. Furthermore, *Rf5* and *Rf1a* were found to be the same gene, and the *Rf1a(Rf5)*/*Rf1b* locus is closely linked to *Rf4* on chromosome 10.

Currently, three-line *indica* hybrids are widely used, but the shortcomings of BT-type CMS (unstable sterility of some BT-type CMS lines and the threat of genetic vulnerability when using a single cytoplasm source) have restricted its use, and thus the cultivation area of three-line *japonica* hybrids relative to the entire *japonica* rice planting area in China is only ~ 5% (Deng et al. 2006). Most rice breeders are of the opinion that accelerating the development and application of *japonica* hybrids may be an effective way to increase the total rice grain yield in China. Considering the successful use of WA-type CMS and the stable sterility of WA-type CMS lines in *indica*-type cultivars, breeders have attempted to use the WA-type CMS in *japonica* hybrids; however, they encountered several problems such as difficulty in obtaining restorer lines and deteriorating flowering habits of WA-type *japonica* CMS lines (Tang et al. 2008; Yang 1994). Thus, WA-type CMS has no practical use in *japonica* rice production, and there are few studies that have examined the genetic basis of fertility restoration in WA-type *japonica* CMS lines*.* In breeding, the *japonica* restorer lines used in China have been only bred for BT-type *japonica* CMS, and most restorers usually carry the *Rf1* locus (Akagi et al. 1996; Chen and Liu 2014; Huang et al. 2014; Komori et al. 2004; Wang et al. 2006; Yang et al. 2016). *Rf1* used for breeding of BT-type CMS restorer lines in China is first transferred from ‘IR8’, which is a donor of *Rf* genes to ‘IR24’, a known *indica* restorer for WA-type *indica* CMS lines carrying *Rf4*, and this *Rf1* resource has been used in most BT-type *japonica* restorer lines (Yang et al. 2016). It seems reasonable to assume that most BT-type *japonica* restorer lines should carry *Rf4*. However, this is confusing, because BT-type *japonica* restorers usually exhibit a poor ability to restore male fertility in WA-type *japonica* CMS lines (Zhu et al. 2010; Zhang et al. 2018). Therefore, it is important that we understand the genetic basis of fertility restoration in WA-type *japonica* CMS lines, which will be helpful for breeding high-yielding WA-type *japonica* rice hybrids.

In breeding practice, C418 is an elite BT-type restorer line that has been widely used for breeding *japonica* hybrids in China. Here, we report that C418 is able to restore fertility in WA-type *japonica* CMS lines, and demonstrate that *Rf4* is present in C418 and is responsible for the fertility restoration. Furthermore, we analyzed the functional model and effects of *Rf4* on fertility restoration in WA-type *japonica* CMS lines. The results of our study will assist breeders in analyzing the differences in fertility restoration in WA-type CMS in *indica*/*japonica* genetic backgrounds and will strengthen the breeding of *japonica* restorer lines for the development of WA-type *japonica* hybrids.

## Materials and Methods

### Plant Materials

Two WA-type *japonica* CMS lines, ‘WA-LiuqianxinA’ (WA-LqxA) and ‘WA-NipponbareA’ (WA-NipA), and the *japonica* restorer line C418 were used in this study. To evaluate the fertility restoration ability of C418 in WA-type *japonica* CMS lines, two testcross F_1_ hybrids were generated by separately crossing C418 with WA-LqxA and WA-NipA. To identify the *Rf* locus involved in the fertility restoration of WA-type CMS, the WA-NipA//Nip/C418, WA-NipA/C418 F_2_, and WA-LqxA/C418 F_2_ populations were constructed for the genetic analysis of fertility restoration and gene mapping. To analyze the vigor of pollen grains produced by the testcross F_1_ plants, the WA-NipA//WA-NipA /C418 population was generated. We crossed plants harboring the heterozygous alleles of *Rf4* in the Nip//Nip/C418 population, followed by four backcrosses to Nip, and the near-isogenic line for *Rf4* (NIL^*Rf4*^) was developed from the BC_5_F_3_ population via molecular marker-assisted selection. For evaluation of the *Rf4* gene’s ability to restore fertility to WA-type *japonica* CMS lines, we made two testcross F_1_ hybrids, WA-NipA/NIL^*Rf4*^ and WA-LqxA/NIL^*Rf4*^*,* and generated the WA-NipA/NIL^*Rf4*^//NIL^*Rf4*^ population. All of these materials were planted in the experimental field at Yangzhou University in Yangzhou, Jiangsu Province, China.

### Fertility Scoring and Genetic Analysis

The pollen grain fertility and natural spikelet fertility of five plants from the CMS lines, 10 plants from the testcross F_1_ lines, and each plant in the WA-NipA//Nip/C418, WA-NipA/C418 F_2_, WA-LqxA/C418 F_2_, WA-NipA//WA-NipA/C418, and WA-NipA/NIL^*Rf4*^//NIL^*Rf4*^ populations were observed. For pollen grain fertility, three mature anthers were harvested and the pollen grains were stained with 1% I_2_–KI solution. Thus, the number of dark-blue, clear (unstained), and typical abortive pollen grains in each individual were counted under a microscope, and the pollen grain fertility was estimated using the percentages of stained pollen grains. Natural spikelet fertility levels were measured as the average seed-setting rates by counting the filled and unfilled grains on the main panicle of one plant harvested 20 days after flowering.

For the genetic analysis, the stained pollen grain rate was used as the main criterion for the evaluation of sterile and partially fertile plants. Plants with a typical aborted pollen grain rate of > 95% were categorized as sterile, and those with a > 90% stained pollen grain rate were considered to be partially fertile. A chi-squared test was used to test the goodness-of-fit of the hypothesis.

### DNA Extraction, PCR Amplification, and DNA Sequencing

Total genomic DNA was extracted from fresh leaves using the cetyltrimethylammonium bromide (CTAB) method with some modifications (Rogers and Bendich 1985). STS10-27 and STS10-16, two insertion/deletion markers that map to loci that are closely linked to the *Rf4* gene, were used to genotype the individuals in the fertility segregation populations. The molecular marker amplifications were performed in 20 µL reactions containing 1 × supplied PCR buffer, 0.1 mmol/L of each dNTP, 1.0 U *Taq* polymerase, 0.2 µmol/L primer, and 20 ng template DNA. Amplification was performed using the following procedure: samples were denatured at 94 °C for 4 min, followed by 32 cycles of 94 °C for 45 s, 55 °C for 45 s, and 72 °C for 50 s, with a final extension step at 72 °C for 5 min. The amplification products were separated by electrophoresis in 3% agarose gels containing ethidium bromide, and the bands were visualized with a GEL DOC 1000 system (Bio-Rad Company, Hercules, CA, USA). DNA fragments covering *Rf4* from ‘C418’ were amplified using high-fidelity GXL-*Taq* (Well-Offer Biotechnology Company, Nanjing, China). The analyzed sequences contained the 206-bp 5′-upstream region and the 164-bp 3′-downstream region of the *Rf4* gene. PCR products were recycled using a recycling kit (Vazyme, Nanjing, China), and then ligated into the *pEASY*-Blunt Zero vector. Five plasmids were sequenced by GENEWIZ (Suzhou, China), and a clone with the correct DNA sequence was selected. A sequence alignment was performed with the BLAST algorithm-based network services at the National Center for Biotechnology Information (NCBI). The names and DNA sequences of the primers used in this study are given in Additional file 1: Table S1.

**Table 1:** Fertility segregation in the F_2_ populations and three-cross F_1_ progeny plants

Population	Total plants	Partially fertile plants	Sterile plants	Observed ratio
WA-NipA//Nip/C418	160	88	72	1.22:1
WA-NipA/C418 F_2_	350	343	7	49:1
WA-LqxA/C418 F_2_	254	251	3	83.67:1

The genetic background of NIL^*Rf4*^ was determined with the 40 k rice SNP-array, a whole-genome single nucleotide polymorphism (SNP) array with 40,000 SNP and InDel markers (Greenfafa, Wuhan).

### Data Analysis

The analysis of variance (ANOVA) package in SPSS15.0 was used for statistical analysis of the fertility of the lines and populations used in this study.

## Results

### Evaluation of the Fertility Restoration Capability of C418

**Fig. 1:**
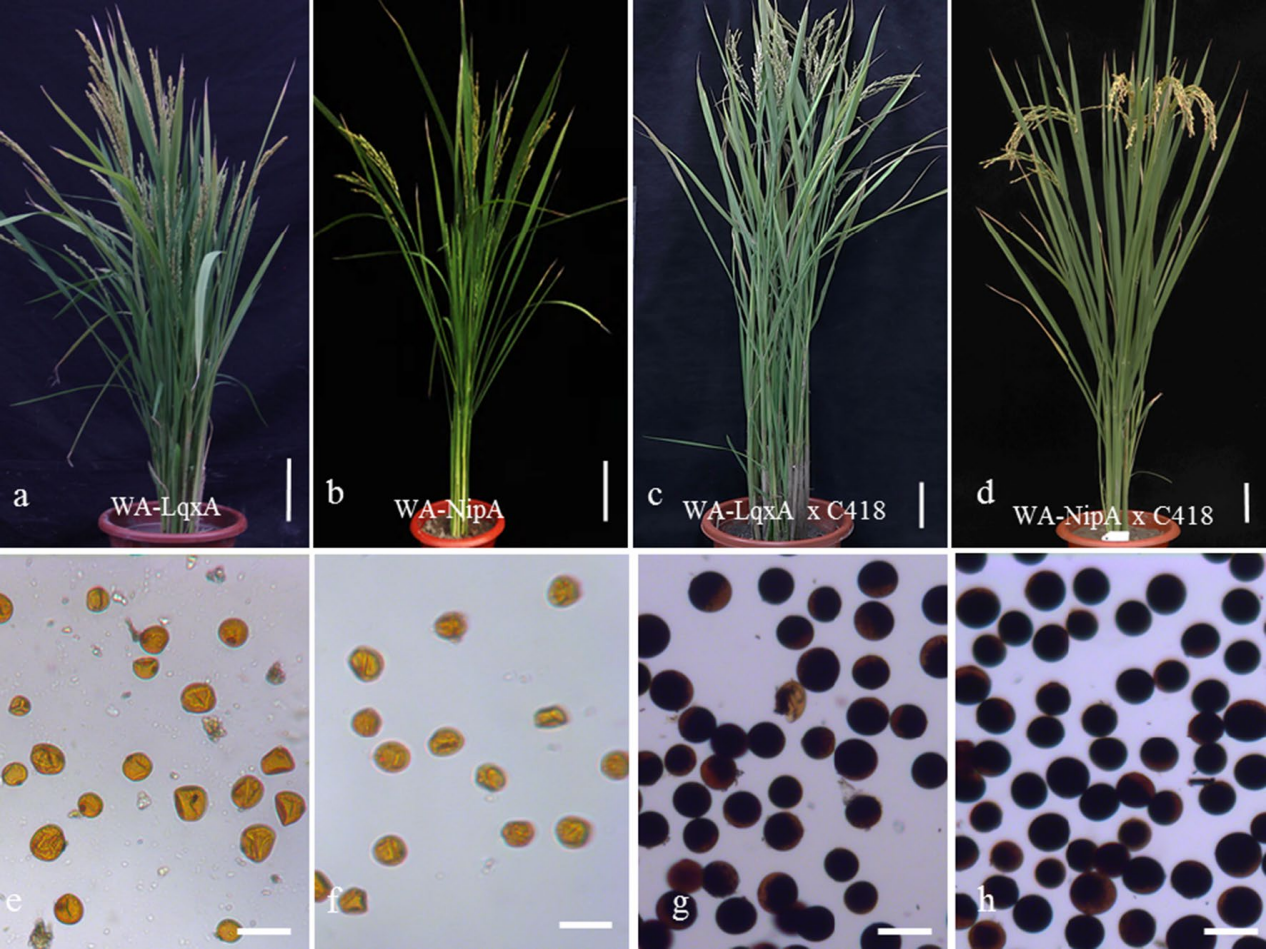
Gross morphology of mature rice plants and pollen viability. **a** ‘WA-LiuqianxinA’ (WA-LqxA). **b** ‘WA-NipponbareA’ (WA-NipA). **c**, **d** F_1_ plants of WA-LqxA × C418, and WA-NipA × C418. **e**–**h** Pollen grains stained with I_2_–KI from corresponding plants of WA-LqxA, WA-NipA, WA-LqxA × C418 F_1_ and WA-NipA × C418 F_1_, respectively. Scale bars = 20 cm in **a**–**d**, 50 μm in **e**–**h**

To examine the ability of C418 to restore fertility in WA-type *japonica* CMS lines, we determined the pollen grain and spikelet fertility levels of individual plants of two WA-CMS lines and the testcross F_1_ hybrids obtained from the crosses between two WA-type CMS lines and C418. WA-NipA and WA-LqxA exhibited amorphous aborted pollen grains, and the natural spikelet levels of these two CMS lines were 0, indicating that the WA-type *japonica* CMS lines were completely sterile (Fig. 1a, b, e, f). The pollen stainability of the testcross F_1_ plants was > 95%, and the natural spikelet fertility levels of these testcross F_1_ plants ranged from 10.86 to 64.63%, indicating that plants carrying the WA-type CMS cytoplasm produced morphologically normal pollen, although partially stained pollen grains may lack the ability to germinate (Fig. 1). Also, the average natural spikelet fertilities of the WA-LqxA/C418 F_1_ and WA-NipA/C418 F_1_ plants were statistically different (24.63% ± 7.70% vs. 54.06% ± 8.47%, P = 4.24E−6). These results indicate that the restorer line C418 is able to partially restore fertility to the WA-type *japonica* CMS lines tested in this study, and that fertility restoration was influenced by the subtypes of the CMS line nuclear backgrounds.

### Genetic Analysis of Fertility Restoration and Gene Mapping

In order to explore the mechanism underlying fertility restoration, the pollen grains and natural spikelet fertility levels of 160 plants in the WA-NipA//Nip/C418 population were investigated. Based on the pollen grains, the plants in the WA-NipA//Nip/C418 population could be divided into two categories: (1) the plants resembled the CMS lines with degenerated anthers and shrunken pollen grains and were sterile, and (2) the plants were similar to the testcross F_1_ hybrids with normal anthers and dark-stained pollen grains and were partially fertile. The segregation ratio of sterile plants to partially fertile plants was 1:1 (*χ*^2^ = 0.21, which was < *χ*^2^_0.05_ = 3.84), indicating that fertility restoration is conditioned by one dominant restorer gene (Table 1). The natural spikelet fertilities of the sterile plants were < 3.88%, and the natural spikelet fertilities of the partially fertile plants ranged from 0 to 61.92% and showed a continuous variation. These results indicate that there might be one or more minor-effect *Rf* genes in C418 that affect fertility restoration in WA-type CMS lines.

**Fig. 2:**
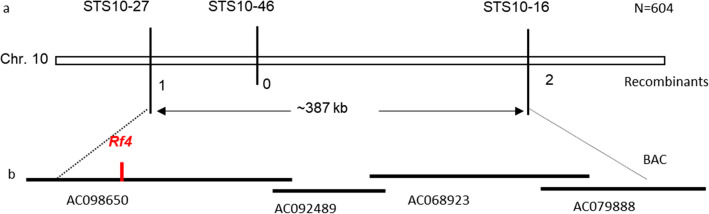
Genetic mapping of the *Rf* gene. **a** *Rf* gene was mapped into ~ 387 kb physical region flanked by marker STS10-27 and STS10-16 on chromosome 10 that overlapped by AC068950, AC092489, AC068923 and AC079888 (**b**)

Based on the breeding pedigrees of BT-type *japonica* hybrids in China, most BT-type restorer lines should theoretically carry the major restorer gene *Rf4* (Yang et al. 2016; Kazama and Toriyama 2014). In order to map the target *Rf* gene in C418, we first needed to determine whether *Rf4* is present in C418. We sequenced the *Rf4* allele from C418, and the nucleotide sequence of *Rf4* in the C418 was identical to that from IR24, demonstrating that it carried the functional *Rf4* allele. Subsequently, we tested whether *Rf4* is related to the fertility restoration of WA-type CMS by genetic linkage analysis. A previous study identified two InDel marker loci, STS10-27 and STS10-16, that flank *Rf1*/*Rf4* on Chromosome10, and these markers show polymorphisms between C418 and Nip. We used these two markers to genotype all plants in the WA-NipA//Nip/C418 population. All 88 partially fertile plants showed heterozygous genotypes at these two loci, while all 72 sterile plants were homozygous for the NIP alleles at the two marker loci. Meanwhile, three recombinants were identified. Based on the genotype and phenotype of recombinants, *Rf* gene was mapped in the physical region of ~ 387 kb co-segregated with molecular marker STS10-46, where *Rf4* was contained (Fig. 2). These results indicate that the *Rf* gene in C418 that is responsible for the fertility restoration of WA-type CMS is most probably *Rf4*.

### The ***Rf4*** Pollen Grains are Favored in Fertilization in the Testcross F_1_ Plants

A total of 350 and 254 plants in the WA-NipA/C418 F_2_ and WA-LqxA/C418 F_2_ populations, respectively, were grown to maturity, and the pollen grains and natural spikelet fertilities of all F_2_ plants were quantified. Based on the fertility criteria (Materials and methods), there were 343 and 251 partially fertile plants, and seven and three sterile plants in the WA-NipA/C418 F_2_ and WA-LqxA/C418 F_2_ populations, respectively (Table 1). These results indicate that the fertility restoration in WA-type CMS *japonica* rice is sporophytic, but the segregation ratio of partially-fertile plants to sterile plants did not fit a one-gene (3:1) sporophytic model. To explore the reasons behind the aberrant segregation of fertility restoration, we used the markers STS10-27 and STS10-16 to genotype all of the F_2_ plants. In the WA-NipA/C418 F_2_ population, there were 192 plants with the *Rf4Rf4* genotype and 151 plants with the *Rf4rf4* genotype*,* all of which were partially fertile, while the seven sterile plants had the *rf4rf4* genotype. Similarly, 251 partially fertile plants in the WA-LqxA/C418 F_2_ population were *Rf4*-containing plants, including 137 plants with the *Rf4rf4* genotype and 114 plants with the *Rf4Rf4* genotype, and three sterile plants carried the *rf4rf4* genotype. Obviously, the genotypes at the *Rf4* locus deviated from the expected 1:2:1 segregation ratio in the two F_2_ populations for a common single gene model. However, plant phenotypes were completely consistent with their genotypes, indicating that *Rf4* is indeed correlated with the fertility restoration of WA-type CMS.

In considering the aberrant fertility and genotypic segregation ratios, we hypothesized that both *Rf4* and *rf4* pollen grains in the testcross F_1_ plants are able to restore fertility in WA-type *japonica* CMS lines, but that the *rf4* pollen grains are less competitive than the grains carrying *Rf4*. To test this hypothesis, we further constructed the WA-NipA//WA-NipA/C418 population that consisted of 37 plants, and the pollen grains and genotypes of these plants were investigated. We found that 35 plants had > 90% stained pollen grains and the *Rf4rf4* genotype, while two plants showed > 95% shrunken pollens and had the *rf4rf4* genotype. This distorted segregation indicated that the majority of male gametes in the WA-NipA/C418 F_1_ plants involved in pollination should carry the *Rf4* gene. Taken together, these results demonstrate that the pollen grains carrying *Rf4* in the testcross F_1_ plants have different viability compared to grains that do not carry *Rf4*, and that *Rf4* pollen grains are preferentially selected during fertilization.

### Development of NIL^***Rf4***^ in the ‘Nipponbare’ ***Japonica*** Genetic Background

**Fig. 3:**
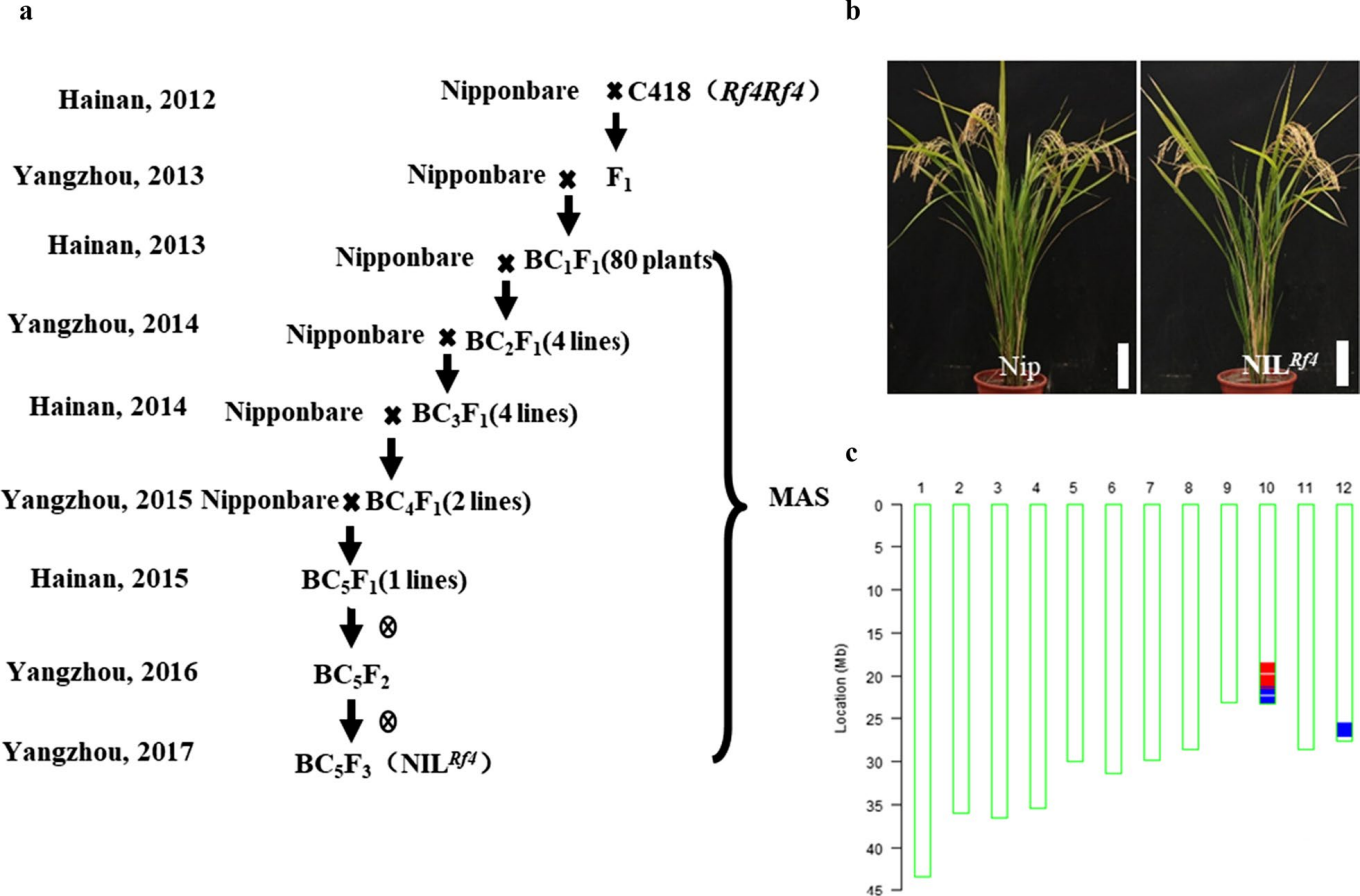
Breeding scheme and gross plant morphology of the parental line and the newly-developed NIL (near-isogenic line) in the ‘Nipponbare’ genetic background. **a** Breeding scheme showing the development of NIL^*Rf4*^. **b** Mature plants of ‘Nipponbare’ and NIL^*Rf4*^. Scale bars = 20 cm. **c** Genomic constitution of NIL^*Rf4*^. The red and blue boxes on the chromosomes indicate the introgressed chromosomal segments derived from ‘C418’; the red box represents a region that is homozygous for the C418 genotype, and the blue boxes represent heterozygous regions. The substituted regions on the distal end of Chr. 10 cover the region in which *Rf4* is located

In an attempt to identify the effects of *Rf4* on the fertility restoration of WA-type CMS in *japonica* lines, we developed a near-isogenic line homozygous for *Rf4* in the Nip genetic background using marker-assisted selection (Fig. 3a). In 2017, a BC_5_F_3_ line was obtained that was very similar to Nip in terms of its main biological characteristics (Fig. 3b). In addition, the genetic background of the BC_5_F_3_ line was analyzed using the 40 K rice SNP-array, which showed that the recurrent parent genome recovery was 98.45% in this line, and the C418 chromosome segment containing *Rf4* had been successfully transferred into Nip. Thus, we successfully developed an *Rf4* NIL*,* which we named NIL^*Rf4*^*,* and it was suitable for further study.

### The Effects of *Rf4 *on the Fertility Restoration of WA-type *Japonica* CMS Lines

**Fig. 4:**
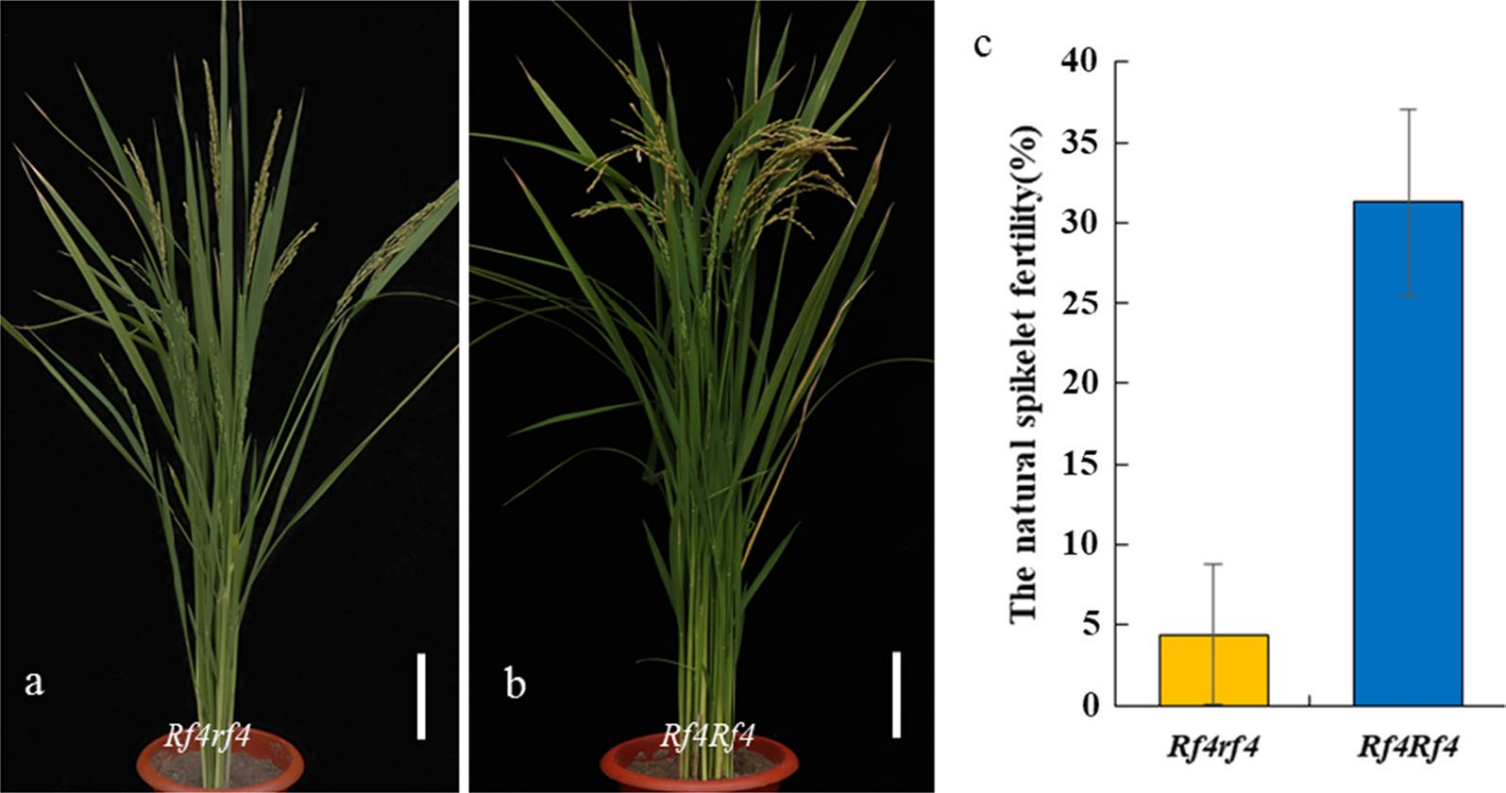
Natural fertility levels of plants with different *Rf* genotypes in the WA-NipA/NIL^*Rf4*^//NIL^*Rf4*^ population. **a** Mature plants with the *Rf4rf4* genotype. **b** Mature plants with the *Rf4Rf4* genotype. **c** Natural spikelet fertility levels of the WA-NipA/NIL^*Rf4*^//NIL^*Rf4*^ plants. Scale bars = 20 cm in **a** and **b**

We next examined the pollen grains following I_2_-KI staining and determined the natural spikelet fertility levels of WA-NipA/NIL^*Rf4*^ F_1_ and WA-LqxA/NIL^*Rf4*^ F_1_ plants. All F_1_ plants had dark-staining pollen grains, and there were no differences between the different crosses (data not shown). The natural spikelet fertility levels of the testcross F_1_ hybrids from WA-NipA and NIL^*Rf4*^, and WA-LQXA and NIL^*Rf4*^ were all < 1%. These results indicate that *Rf4* has minor effects on the fertility restoration of WA-type *japonica* CMS lines. In the WA-NipA/NIL^*Rf4*^//NIL^*Rf4*^ population, there were 14 plants with the *Rf4rf4* genotype and 19 plants with the *Rf4Rf4* genotype, and the average natural spikelet fertility levels of the plants were 4.40% and 31.32%, respectively (Fig. 4). These results imply that *Rf4* exerts a dosage effect on the fertility restoration of WA-type *japonica* CMS lines.

## Discussion

Wild-abortive (WA) CMS is an ideal type of sporophytic CMS that has been used for the large-scale commercial production of hybrid *indica* rice since the 1970’s in China (Barclay 2010; Li et al. 2007). However, insufficient numbers of *japonica* restorer lines have been a major limiting factor in the application of WA-type CMS to hybrid production of *japonica* rice seed. The breeding of WA-type *japonica* restorer lines has been a crucial issue in the development of WA-type *japonica* hybrids. In the *japonica* subspecies, only BT cytoplasm has been used for seed production, and the restorer lines have been bred for BT-type CMS lines. There are two ways to generate WA-type *japonica* restorer lines; the first is to systematically search for WA-type *japonica* restorer lines in the BT-type *japonica* restorer lines by testcrossing, and the second is to breed new WA-type *japonica* restorer lines. In our previous study, most BT-type restorer lines were able to restore fertility in WA-type *japonica* CMS lines, but most of the testcross F_1_ plants had low spikelet fertility rates (Zhu et al. 2010; Zhang et al. 2018). It is difficult to search for WA-type *japonica* restorer lines in the existing BT-type *japonica* restorer lines, but is a reasonable strategy to breed new WA-type *japonica* restorer lines based on BT-type *japonica* restorer lines. Thus, understanding the genetic basis of fertility restoration in WA-type *japonica* CMS lines and the *Rf* genes that are present in BT-type restorer lines will assist in breeding of WA-type *japonica* restorer lines. Based on the breeding process of three-line *japonica* hybrids, C418, a leading BT-type *japonica* restorer line with a partial *indica* phenotype, has been used widely for breeding three-line hybrids in China (Yang et al. 2016). Thus, we considered C418 to be a suitable parent to use in a study of the genetic basis of fertility restoration in WA-type *japonica* CMS lines and to identify the *Rf* genes that are important for fertility restoration of WA-type CMS in BT-type *japonica* restorer lines. In the present study, we found that C418 can restore the fertility of WA-type CMS in testcross F_1_ plants, which is consistent with the results of our previous study (Zhang et al. 2018). Many studies have shown that fertility restoration is influenced by genetic background, environmental factors (such as temperature and relative humidity), and the interaction with the different types of CMS cytoplasm. We found variable spikelet fertility levels in the testcross F_1_ hybrids derived from C418. Considering that the testcross F_1_ hybrids exhibited similar heading dates (data not shown), we suggest that the differences in the ability of C418 to restore fertility in the CMS lines could be related to the existence of unknown genes that participate in the fertility restoration of WA-type CMS in the WA-type *japonica* CMS lines.

It is well known that fertility restoration of WA-type CMS in *indica* lines is mainly controlled by two major *Rf* genes, *Rf3* and *Rf4*. However, pollen and spikelet fertility levels usually show a continuous distribution in a given genetic population, and different genetic hypotheses have been suggested for fertility restoration in WA-type CMS in previous studies (Govinda Raj and Virmani 1988; Bharaj et al. 1991). In the present study, a dominant gene in C418 was found to restore normal anthers and stainable pollen grains to WA-type *japonica* CMS lines. Through DNA sequencing and linkage analysis, the *Rf4* gene in C418 was identified as being responsible for fertility restoration of WA-type CMS. However, continuous variation in spikelet fertility levels of the partially fertile plants was observed in the three-cross population, indicating that there might be minor-effect *Rf* genes in C418 which can affect the fertility restoration of WA-type CMS. Also, the testcross F_1_ plants derived from WA-LqxA and C418 in this study had stainable pollen grains and natural spikelet fertility levels of 20%, but the testcross F_1_ plants derived from WA-LqxA and C9083, a BT-type *japonica* restorer carrying *Rf4*, exhibited typical abortive pollens and no natural spikelet fertility in our previous study, which provides additional evidence that there are other *Rf* genes in addition to *Rf4* in C418 (Zhang et al. 2018). Also, the natural spikelet fertilities of WA-NipA/NIL^*Rf4*^ F_1_ plants were significantly lower than in the WA-NipA/C418 F_1_ plants, providing yet another piece of evidence to show that C418 carries other *Rf* genes as well as *Rf4*. To identify the unknown *Rf* genes in C418, we are constructing chromosome segment substitution lines from the cross of C418 (the recipient) and Nip (the donor), which will lay a solid foundation to discover the genetic basis of fertility restoration of WA-type CMS in *japonica* rice lines.

Fertility restoration of WA-type CMS has been documented as a sporophytic mechanism because *indica* rice plants with the *Rfrf* genotype and CMS produce all fertile pollen grains. The pollen grains produced in the testcross F_1_ hybrids are thought to transmit the *Rf* and *rf* alleles with the same frequency through the paternal parent. As a result, sterile plants with the *rfrf* genotype can be observed in F_2_ populations, and the percentage of sterile plants is approximately 25% in the single-locus model. In the present study, the appearance of male-sterile individuals in the F_2_ populations derived from WA-type CMS *japonica* lines and C418 indicates that *Rf4* sporophytically restores male fertility, but the percentage of sterile plants was extremely low, deviating drastically from the expected 1:4 ratio in normal sporophytic fertility restoration. Furthermore, the ratios of plants with the *Rf4Rf4*, *Rf4rf4*, and *rf4rf4* genotypes in these F_2_ populations deviated from the expected 1:2:1 ratio in the sporophytic model, and the ratios of partially fertile plants with the *Rf4Rf4* and *Rf4rf4* genotypes were similar to a gametophytic one-gene 1:1 segregation pattern. Based on the genotypes and phenotypes of plants in the WA-NipA//WA-NipA/C418 population, we were able to confirm that the *rf4* pollen grains are much less competitive than the *Rf4* pollen grains in the testcross F_1_ plants, and most of the *rf4* grains may fail in fertilization due to lack of viability or reduced competitiveness relative to the *Rf4* pollen grains. Thus, the results of our study are the first to show that pollen grains carrying *Rf4* are functionally favored in fertilization over pollen grains that carry *rf4* in the presence of WA-type cytoplasm in the *japonica* genetic background, which differs from the functional model of *Rf4* acts to restore male fertility in the *indica* genetic background. Further studies will be required to identify factors that affect the viability of *rf4* pollen grains in the *japonica* genetic background, which will bring new insights on the genetic basis of fertility restoration in WA-type CMS.

In WA-type *indica* hybrids, the two major genes *Rf3* and *Rf4* have been required for full fertility restoration capabilities of WA-type *indica* CMS lines, and these two genes exert an additive effect on fertility restoration (Ahmadikhah and Karlov 2006; Tang et al. 2014; Zhang et al. 1997, 2002). Also, the ability of *Rf4* to restore fertility is greater than that of *Rf3,* and *Rf4* is able to restore a high level of spikelet fertility to WA-type CMS lines (Cai et al. 2013; Zhang et al. 2002; Yao et al. 1997). In the present study, the natural spikelet fertilities of WA-LqxA/NIL^*Rf4*^ F_1_ and WA-NipA/NIL^*Rf4*^ F_1_ plants were very low (< 1%). Thus, our results confirm that the capability of *Rf4* to restore fertility in WA-type *japonica* CMS lines is poor, which is consistent with our previous results and also provides a good explanation of why most BT-type *japonica* restorer lines are not good at restoring the fertility of WA-type *japonica* CMS lines (Tada 2007; Zhang et al. 2018). However, the different fertility restoration capability of *Rf4* in the *indica*/*japonica* genetic backgrounds indicates that minor-effect *Rf* genes are widely present in WA-type *indica* CMS lines, and that these genes can modify the effects of *Rf4* on the fertility restoration of WA-type CMS. In the WA-NipA/NIL^*Rf4*^//NIL^*Rf4*^ population, the average fertility levels of plants carrying the *Rf4Rf4* genotype were greater than those of plants carrying the heterozygous *Rf4rf4* genotype, indicating that the dosage of the *Rf* gene influences the fertility restoration of WA-type CMS *japonica* lines. Additionally, the *Rf4Rf4* plants had natural spikelet fertilities of only 31.32%, indicating that it is not feasible to breed the WA-type *japonica* restorer lines only based on *Rf3* and *Rf4*. Thus, exploring other *Rf* genes for WA-type CMS is crucial to develop WA-type *japonica* restorer lines.

## Conclusion

In summary, we found that the *Rf4* gene is present in C418 and is associated with the fertility restoration of WA-type *japonica* CMS lines. The *Rf4* pollen grains produced by testcross F_1_ plants have priority over the *rf4* pollens in fertilization, although *Rf4* restores male fertility in a sporophytic manner. *Rf4* had minor effects on fertility restoration of WA-type CMS in *japonica* line, and there was a dosage effect of *Rf* on the fertility restoration of WA-type CMS lines. These results will be valuable in future breeding of WA-type *japonica* restorer lines.

### Supplementary Information

**Additional file 1**. **Table S1**: Markers and primers used for gene mapping and sequencing.

## Data Availability

The data that support the findings of this study are available from the corresponding author upon reasonable request.
