# Supplementary material for: The Effects of Rf4 and the Genetic Mechanism Behind Fertility Restoration of Wild Abortive Cytoplasmic Male Sterility (WA-CMS) in Japonica Rice (Oryza sativa ssp. Japonica)
Source: Rice (N Y). 2022 Nov 28;15:59. doi: 10.1186/s12284-022-00605-0 (PMC9705664; doi:10.1186/s12284-022-00605-0)
Supplement: Supplementary file 1 — Additional file 1. Table S1: Markers and primers used for gene mapping and sequencing. [file 12284_2022_605_MOESM1_ESM.docx]

**Supplementary Materials**

**The effects of *Rf4* and the genetic mechanism behind fertility restoration of wild abortive cytoplasmic male sterility (WA-CMS) in *japonica* rice (*Oryza sativa* ssp. *japonica*)**

Honggen Zhang^1, 2^*, Xixu Li ^1^, Zuopeng Xu^1^, Xiangqiang Zhao^3^, Zihao Wan^1^, Xiaojun Cheng^1^, Qiaoquan Liu^1, 2^, Minghong Gu^1^, Shuzhu Tang^1, 2^*

1) Jiangsu Key Laboratory of Crop Genetics and Physiology/ Key Laboratory of Plant Functional Genomics of the Ministry of Education/ Jiangsu Key Laboratory of Crop Genomics and Molecular Breeding, Agricultural College of Yangzhou University, Yangzhou 225009, China

2) Jiangsu Co-Innovation Center for Modern Production Technology of Grain Crops, Yangzhou University, Yangzhou 225009, China

3) School of Life Sciences, Nantong University

*H. Zhang, X. Li and Z. Xu contributed equally to this work.*

*E-mail: [zhg@yzu.edu.cn](mailto:zhg@yzu.edu.cn); [sztang@yzu.edu.cn](mailto:sztang@yzu.edu.cn)

Tel: +86-514-87972148

Supplemental Table 1. Markers and primers used for gene mapping and sequencing

| Primer | Forward Primer (5′-3′) | Reverse Primer (5′-3′) | Purpose |
| --- | --- | --- | --- |
| STS10-27 | GCATTGGAATCAGTGTATCA | TAATGATAGATAGCAGATGT | Mapping |
| STS10-16 | CAGCAATCGGATCGCCTC | ATCTTTCCGTGATGGGAGGTA | Mapping |
| STS10-46 | GGAGCGTTTGTTTAGTTC | TTGCCTATTCCCATGTAC | Mapping |
| *Rf4-1* | TGAGGTGATCTGCTTGCTA | GCTGAAGTTGGAGTTGGA | Sequencing |
